# Memcached: An Experimental Study of DDoS Attacks for the Wellbeing of IoT Applications

**DOI:** 10.3390/s21238071

**Published:** 2021-12-02

**Authors:** Nivedita Mishra, Sharnil Pandya, Chirag Patel, Nagaraj Cholli, Kirit Modi, Pooja Shah, Madhuri Chopade, Sudha Patel, Ketan Kotecha

**Affiliations:** 1 Symbiosis Institute of Technology, Symbiosis International (Deemed) University, Pune 412115, India; nivedita.mishra.phd2019@sitpune.edu.in; 2 Computer Science & Engineering, DEPSTAR, Anand 388421, India; chiragpatel.dce@charusat.ac.in; 3 Department of Information Science and Engineering, R. V. College of Engineering, Banglore 560059, India; Nagaraj.cholli@rvce.edu.in; 4 Sankalchand Patel College of Engineering, Sankalchand Patel University, Visnagar 384315, India; kjmodi.fet@spu.ac.in; 5 Information Technology Department, Gandhinagar Institute of Technology, Ahmedabad 382010, India; pooja.shah@git.org.in (P.S.); madhuri.chopade@git.org.in (M.C.); 6 SALITR, SAL Campus, Ahmedabad 380060, India; sudha.patel@sal.edu.in; 7 Symbiosis Centre for Applied Artificial Intelligence, Symbiosis International (Deemed) University, Pune 412115, India; drketankotecha@gmail.com

**Keywords:** DDoS attacks, Memcached, amplification attacks, botnet, momentum botnet

## Abstract

Distributed denial-of-service (DDoS) attacks are significant threats to the cyber world because of their potential to quickly bring down victims. Memcached vulnerabilities have been targeted by attackers using DDoS amplification attacks. GitHub and Arbor Networks were the victims of Memcached DDoS attacks with 1.3 Tbps and 1.8 Tbps attack strengths, respectively. The bandwidth amplification factor of nearly 50,000 makes Memcached the deadliest DDoS attack vector to date. In recent times, fellow researchers have made specific efforts to analyze and evaluate Memcached vulnerabilities; however, the solutions provided for security are based on best practices by users and service providers. This study is the first attempt at modifying the architecture of Memcached servers in the context of improving security against DDoS attacks. This study discusses the Memcached protocol, the vulnerabilities associated with it, the future challenges for different IoT applications associated with caches, and the solutions for detecting Memcached DDoS attacks. The proposed solution is a novel identification-pattern mechanism using a threshold scheme for detecting volume-based DDoS attacks. In the undertaken study, the solution acts as a pre-emptive measure for detecting DDoS attacks while maintaining low latency and high throughput.

## 1. Introduction

The Internet of Things (IoT) has achieved a broad reach in terms of its applications in almost every sector of life. In the past, the electronic devices that facilitated various needs were independent, and other isolated systems functioned to meet the required objectives. Since systems such as smart homes [1] are now required to provide the user’s needs remotely, they are prone to cyber attacks, namely, phishing attacks [2], man-in-the-middle attacks, and DDoS attacks [3]. Such IoT devices have scales for generating data, from small-scale applications with a few bytes every second to those with several kilobytes every second, depending upon the addressed application. Various applications associated with the IoT are latency-critical and involve vast amounts of data [4]. Data storage and computation requirements pave the way for cloud computing, fog computing, and edge computing. High reliability with low latency is required for many application scenarios of IoT networks, viz., remote surgery, smart grids, connected vehicles, smart homes, and industrial automation [5]. The cache is used mainly to reduce latency, and vulnerabilities associated with the cache are heavily exploited by DoS attacks, making most IoT applications a target for such attacks.

**Figure 1:**
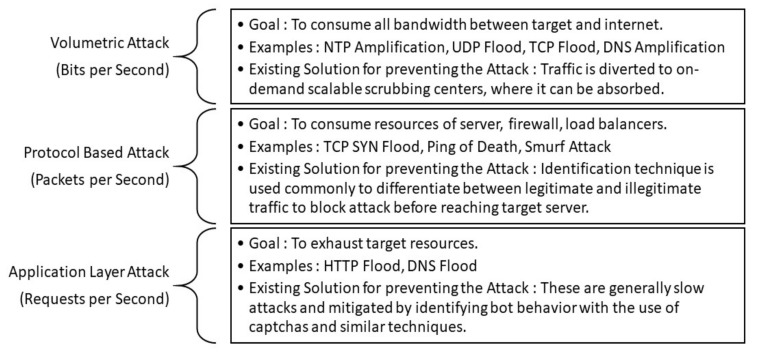
Classification of DDoS attacks.

A denial-of-service (DoS) attack occurs when the attacker’s target is to disrupt the victim’s services by utilizing their resources with the help of forged requests [6]. Distributed denial of services (DDoS) is an amplified DoS attack. In this attack, there are typically many sources from which requests originate, hence the label ‘distributed’. Due to this property, it becomes quite challenging to mitigate DDoS attacks. There are many types of DDoS attacks, namely: TCP SYN flood attacks, teardrop attacks, Smurf attacks, ping-of-death attacks, and botnets. The classification of DDoS attacks is depicted in Figure 1, based on the goal of the attacker and the existing solutions for mitigation of the attack.

DDoS attacks can also be classified as reflection and amplification attacks. In a reflection attack [7], the sizes of the request and the response are the same, whereas in an amplification attack [8], the size of the response is many times bigger than that of the request. An amplification attack is a type of reflection attack with a response that is a multiplied version of the request. The bandwidth amplification factor (BAF) is defined as the approximate number of response bytes sent by an amplifier for a request. The BAF plays a crucial role in defining the severity of an attack. NTP, DNS, and SNMP have widely used protocols for amplification attacks [9]. Lately, some new attack vectors have been used for DDoS attacks, such as Apple remote management services (ARMS), the Ubiquiti discovery protocol, the Constrained Application Protocol (CoAP), Web Services Dynamic Discovery, HTML5 hyperlink-auditing ping redirection, and Memcached. The BAFs of commonly used protocols in DDoS attacks are shown in Figure 1. As is evident from the figure, the BAF of Memcached is several times (≈100 times) greater than that of the other protocols used as DDoS attack vectors [10]. This high BAF led to the analysis of the Memcached protocol in the security context.

**Figure 2:**
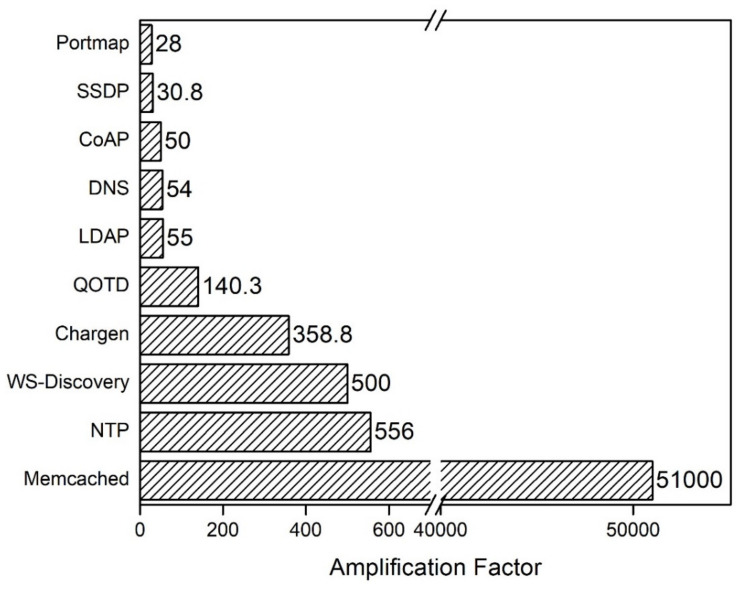
Representation of the bandwidth amplification factor of various protocols.

The Memcached protocol was developed in 2003 [11]. This protocol was introduced to reduce latency, thereby speeding up dynamic web applications by significantly reducing the database load, and is widely used by companies such as Facebook, YouTube, Twitter, and GitHub [12]. The DDoS attack is known for its impact, due to the rate at which it can be initiated/spread. In the past, attackers have launched an attack on Dyn with a strength of 1.2 Tbps. It was considered to be one of the most significant attacks to date, and the malicious actors in the attack were bots. Therefore, botnets were considered a critical aspect of launching significant DDoS attacks; however, after just two years, in 2018, GitHub came under a Memcached DDoS attack which did not involve botnets. The capability of this kind of attack can be understood by the fact that in just a few months, another attack was launched on Arbor Networks with a strength of 1.7 Tbps [13]. The amplification capacity and vulnerabilities of the Memcached protocol raise security concerns. Figure 2 represents the bar-chart representation of various protocols.

In the undertaken study, an architectural change was proposed for detecting DDoS attacks. The significant contributions of the proposed work are:

i. An architectural change was proposed to make Memcached more secure.
ii. The solution acts as a pre-emptive measure for detecting DDoS attacks, thus enhancing system performance at large.
iii. A threshold mechanism was introduced to create an identification pattern for detecting volume-based DDoS attacks, rendering the solution more user-friendly.
iv. A case study for detecting DDoS attacks carried out using a Memcached server was analyzed and discussed.

## 2. Related Work

The Memcached protocol has been used for a long time by large companies such as Facebook and Google to improve the user experience. This protocol is quite useful in web applications. Still, some vulnerabilities need analysis to save cyberspace from significant DDoS attacks due to this protocol in the future. These attacks are possible because UDP services send responses which are much larger than the requests.

Memcached is an open-source, simple, but powerful distributed memory-caching system. Many researchers have worked on different architectures for Memcached servers. Lim et al. [14] introduced thin servers with smart pipes by coupling embedded low-power cores to the Memcached server, making it possible for GET requests to be processed in the hardware, thus reducing the load on the network and database. Memcached is a single-point failure mechanism; Lu et al. [15] addressed this issue and proposed R-Memcached, where caches are replicated in Memcached servers. Blott et al. [16] proposed a hybrid solution by combining DRAMs and serial-attached flash memory. Zaidenberg et al. [17] proposed five new algorithms in place of LRU for Memcached. Memcached can work with single and clustered nodes; Bakar et al. [18] discussed the difference in Memcached servers’ behavior in both scenarios. Cheng et al. [19] analyzed several factors affecting the latency. In addition, they provided essential recommendations, such as reducing the number of keys generated from client requests in place of lowering the cache miss ratio, which is, anyway, very small. Several architectural modifications for Memcached servers are recommended in the literature to decrease the latency and reduce the cost. In Table 1, the architectural changes proposed by researchers in the literature for different problems are discussed. The issues discussed here are related to latency and provide the basis for the proposed work.

High-bandwidth and low-latency networks suffer from incast congestion, where many-to-one traffic patterns are observed, i.e., many clients send data to the same receiver [21]. Web servers tend to communicate with multiple Memcached servers to satisfy a user request in the real world. This leads to communication by web servers with all Memcached servers in a short period, causing incast congestion. With the increase in Memcached servers, the problem of incast congestion increases. The consequence of incast congestion is Memcached error, and as a result, data requests go to the main memory, causing increased latency. Memcached also suffers from cold caches, replication, consistency issues, and rigid scale-out features. Nishtala et al. [12] proposed that these challenges could be dealt with by adapting some trade-offs in the number of Memcached servers, the window size, and the latency.

For data-intensive applications, edge computing is also used. Caching is essential at a network’s edge, and researchers are working on efficient caching techniques [22]. However, without any mechanism, the data stored in the cloud are not accessible in a short period of time. Having many devices connected to the cloud introduces high latency, which is unacceptable in healthcare, firefighting, and similar emergency services [23]. Some Latency-critical IoT applications include factory automation, smart grids, process automation, and intelligent transport systems [24]. Cloud computing offers resilience and reliable services, which is the main reason for its prevalent use in IoT applications. However, the applications assisted by cloud computing are highly prone to DDoS attacks [25]. Caching is a popular technique used to reduce latency in computers. Researchers have proposed several mechanisms for decreasing latency. These include the caching mechanism proposed by Niyato et al. [26] and the middleware for complex-event processing in real-time presented by Baptista et al. [27]. Two-phase load balancing for Memcached shows improvement in both the server and the network load [28]. Chen et al. [29] proposed a cache and prefetching mechanism to decrease the network load and latency by deploying caches at first- and second-tier nodes. IoT applications rely heavily on data; locally storing the data is not advisable. Hence, several techniques are used, such as integrated edge and cloud computing [30]. An IP traffic bandwidth-control policy was presented by Foremski et al. [31] to protect networks. Nishtala et al. [32] developed a hybrid approach for making efficient resource-allocation decisions, utilizing Memcached and web search as back-end services. For each service, the request generator followed a diurnal load pattern. Several architectural changes have been proposed by researchers for the efficient use of Memcached, as seen in the literature. Despite the efforts to improve the architecture of Memcached, only a few research works were conducted in the domain of Memcached. Threshold techniques are predominantly used for discriminating between normal traffic and abnormal traffic [33,34,35,36]. The presented work uses the threshold technique for DDoS attack detection and mitigation in Memcached-based DDoS attacks.

## 3. Cache Attacks and Internet of Things

The non-standardized growth of the IoT industry has been a major contributor to DDoS attacks [37]. With the introduction of IoT devices, there has been a contest to turn everything into a smart format, be it a city or healthcare, agriculture or industry; the IoT is in almost every sector. These devices generate and keep on storing data on scales from small to large, according to the demands of the applications. The industrial IoT (IIoT) has limitless prospects for future industries by analyzing data collected from thousands of connected sensors. The IIoT has several advantages: higher efficiency, improved accuracy, network scalability, predictive maintenance, lesser downtime for machines, power savings, higher security, and optimization. The multiple IoT (MIoT) has vast areas of applicability, one important example being smart cities [4]. The cache is used in the abovementioned IoT applications to reduce latency and increase throughput; this makes IoT applications prone to cache attacks [38]. Memcached is identified in the IoT domain in upcoming industrial applications where the IoT nodes are not limited in terms of the amount of data that can be handled due to an application’s nature. Some scenarios in which IoT applications can become exposed to cache attacks are:

*Smart cities:* Real-time data are a crucial requirement for many applications within a smart city, viz., fire detection and firefighting, water management, lighting, traffic management, and transportation. These kinds of applications are highly critical and require more attention so that security is kept as a priority and the real-time data requirement is met. Sun et al. [39] demonstrated that caching needs to be carried out dynamically to save energy for different IoT applications in smart cities. Naeem et al. [40] projected periodic caching as a solution for latency-critical smart city applications and considered the security requirements. However, cache inconsistency and security are two major problems associated with caching [41].

*Smart grids:* In smart grids, IoT technology is used in smart devices, networking, and communication technologies. The latency involved in a smart grid ranges from milliseconds to subseconds [42]. Yin et al. [43] studied content caching, resource allocation, and energy cooperation in smart grids; this study showed the extensive need for caching techniques in smart grids for using energy efficiently. For processing smart-meter data, communication latency should be lower, and processing rates need to be improved [44]; to achieve this, software platforms are used, which are vulnerable to Memcached attacks.

*Automobile:* In the automobile industry, IoT devices are used for location tracking, the weight measurement of trucks, traffic analysis, and route management. The data collected from these sensors are vast and stored in the cloud; for the real-time analysis of the stored data, some mechanism is required. The Society of Automotive Engineers (SAE) has divided automobile applications into classes A, B, C, and D, with increasing bandwidth, reliability, and stricter latency constraints. The latency requirements vary between 50 ms to 150 ms for class A and 2 ms for class D [45]. Cars connected to the IoT network(CV2X) are called connected cars. In this case, real-time data transmission is also required to increase the driver’s response time. Five levels have been introduced by SAE for vehicle automation, starting from driver assistance to full automation [46]. At level five, very stringent latency is required, along with high reliability. The automotive-maintenance system is also one of the features that uses IoT sensors for predictive analysis. The IoT sensors connected to different parts of a car gather data, and these data are further used to analyze the requirements for the change of the components. Autonomous vehicles are current sensations in the automobile industry, and the purpose of these vehicles is to reduce accidents caused by human error. The IoT systems integrated with these vehicles are used to make real-time decisions, making the caching mechanism a requirement. These applications in the automobile industry can be a hotbed for attacks such as Memcrashed.

*Healthcare:* Latency- sensitive IoT applications in healthcare include medical data collection and the processing of patients, robotic assistance in surgeries, medication delivery, and dispensing prescriptions [47]. Various healthcare applications cannot be accessed if the latency is greater than 200 ms. An application achieving a latency of less than 80 ms is considered suitable for real-time activities [48]. In healthcare systems, DDoS attacks can take place if internet-enabled medical devices are converted to botnets. As they are IoT devices, it is entirely possible to do this, and with the use of amplification attacks, such as Memcrashed, the critical activities of hospitals, such as surgeries, can be controlled [49]. Even worse, if surgeries are in progress and the attacker launches an attack due to insecure IoT devices, several patients’ lives could be in danger.

*Wearables:* IoT technology is used to diagnose health conditions by collecting data for the long term [50]. Wearable devices such as Fitbit smartwatches produce lots of data [51]. While analyzing a sports activity generates, at peak, 25,000 tuples per second of data flow, an even more significant concern is that the minimum flow of data generated is 10,000 tuples per second. This kind of data cannot be stored at the edge, as it is resource-limited; fog computing helps to overcome this issue, as it acts as a bridge between cloud and fog computing. However, the data are kept somewhere in the cache in all of these computing techniques to reduce latency, making these devices vulnerable to cache attacks. Silva et al. [25] described an assessment of solutions based on realistic IoT scenarios, including the flexibility to meet the heterogeneity issues in IoT applications and the mitigation of DDoS attacks using IoT protocols as open research problems in the IoT setup.

## 4. The Necessity of Securing Memcached Architecture

The undertaken study discusses the Memcached protocol widely used in IoT scenarios, and a detection approach for DDoS attacks using Memcached is proposed. Although the Memcached attacks that have been launched to date could be evaded by employing best practices, the attacks’ methodologies are constantly changing; therefore, the vulnerabilities present in Memcached should be addressed. New vulnerabilities can be found out, and they may be used for launching DDoS attacks, as seen in the past. The simple architectural model of Memcached is quite useful for IoT applications; subsequently, Memcached architecture should be made more secure. There have been several cache attacks launched by attackers, the most recent being CPDoS [52]. The most notable cache attack to date was carried out using Memcached, making it our prime focus for further discussion.

### 4.1. Memcached Architecture

Caching is a technique used quite commonly in computing for reducing latency. The cache is a storage space that is used for frequently required entries to save time. Memcached is based on this concept, with some modifications. Memcached is a database-caching system used to speed up websites’ dynamic databases by caching frequent data in DRAM. Memcached uses a key-value method to store data, and it solves the problem of having an extensive data cache [53].

Memcached comprises four components, each of which has a key, an expiration time, and raw data. These components are:

Client: This is a dynamic web-based application that is provided with a list of Memcached servers. One application has several servers associated with it, and only one server at a time receives or shares data. Additionally, the Memcached servers do not share data between themselves.

Client-based hashing algorithm: Typically, there are many clients as well as Memcached servers, so to distribute the load, a hashing algorithm is used to determine which Memcached server should be used. The client chooses the server based on the key.

Server software: The Memcached server calculates the client key’s second hash key to find where to store the key and the corresponding value in the internal hash table.

Least-recently-used (LRU) algorithm: Memcached servers keep data in RAM. For discarding the data, the LRU algorithm is used. This algorithm is quite useful, as it helps to remove old data and keep the memory free for new requirements.

**Figure 3:**
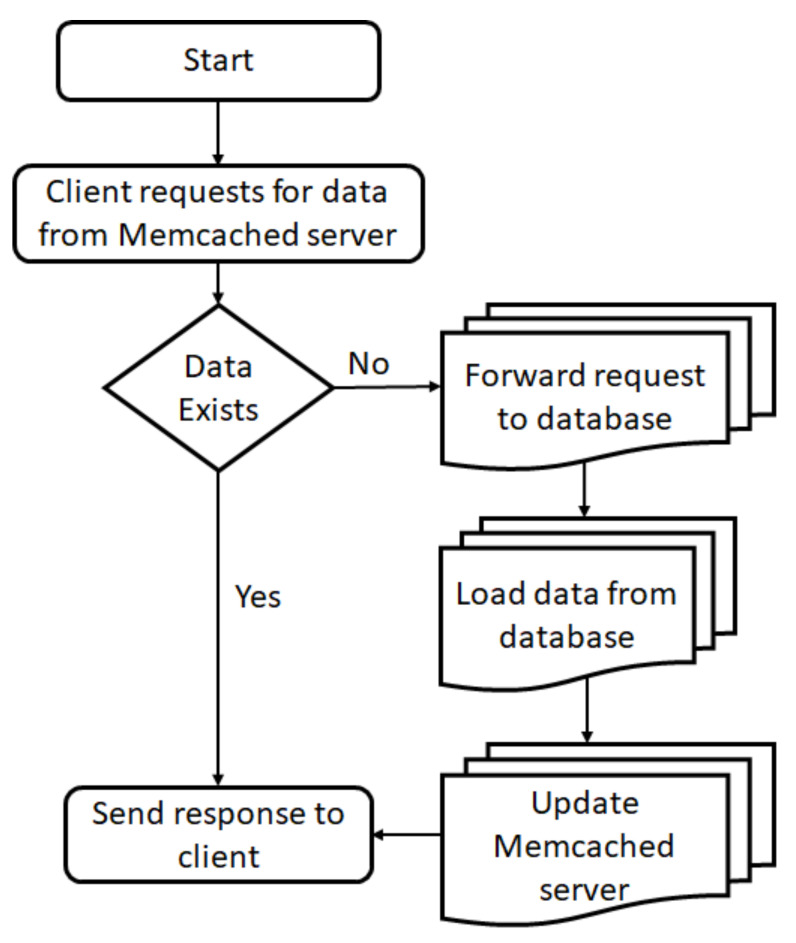
Working of the Memcached server.

As is evident from the flow chart shown in Figure 3, Memcached functions in the following way:

- A client sends a request to the Memcached server for data.
- The Memcached server looks for these data in its cache.
- If the data are present in the cache, the server sends them directly to the client.
- If the data are not present in the cache, then a query is sent to the database, and the retrieved data are saved in the Memcached server and sent to the client.
- If any data are changed or have expired for any value, the Memcached server updates the cache, thus providing updated information to the client.

From this mechanism, it is clear that Memcached reduces the server load, as the inclusion of fewer database calls is required. In addition, standard keys can be stored in the Memcached server to increase the speed of the system. In the case of most probable events, Memcached can be used to enhance performance.

### 4.2. Memcached Attack Mechanism and Case study with Momentum Botnet

**Figure 4:**
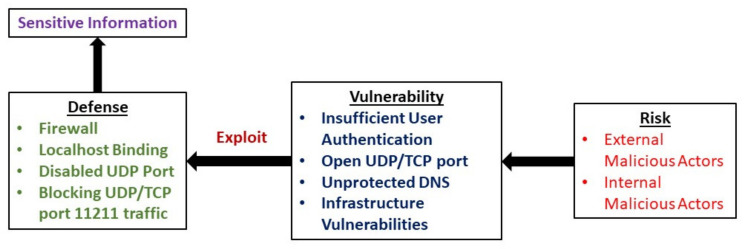
Representation of Memcached attack threat model.

The threat model for Memcached is represented in Figure 4, which describes the potential risk elements and vulnerabilities associated with Memcached and the available defence mechanisms and probable critical assets.

**Figure 5:**
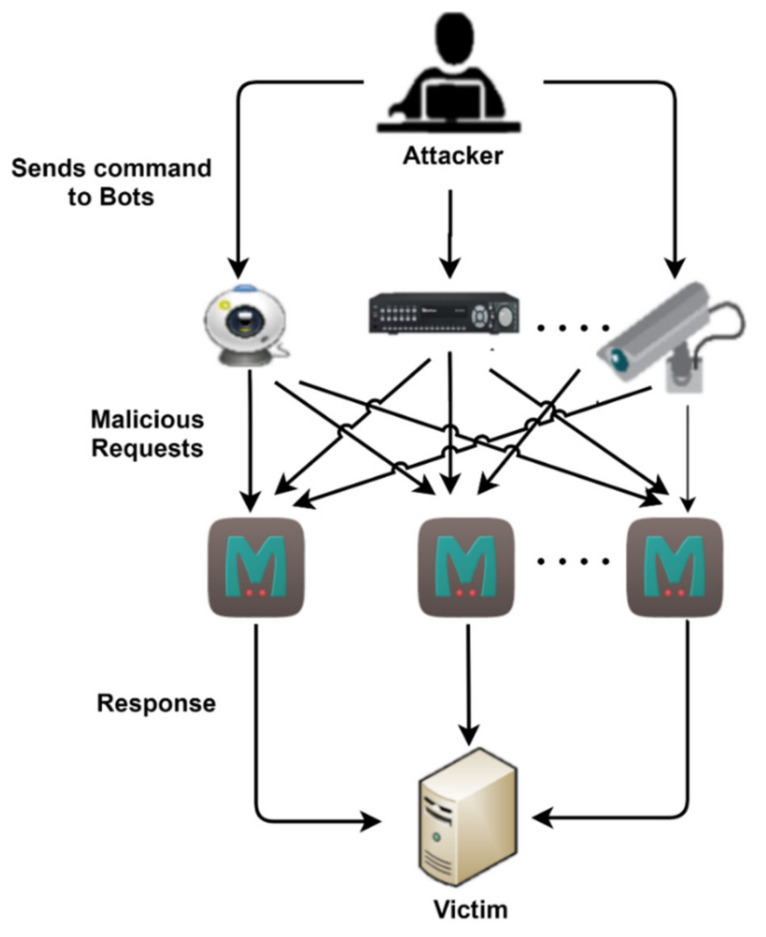
Representation of a Memcached DDoS attack using a botnet.

The Memcached mechanism was designed to work internally, but it became exposed to unauthenticated servers, enabling exploitation via DDoS attacks [53]. A case in which IoT botnets can be deployed is shown in Figure 5, where vulnerable Memcached servers are used for launching attacks. Although patches released after the attack ensured that no attack could occur again using this vulnerability alone if best practices were followed, it could very well be used as an attack vector for botnets. A small IoT bots network deployed for this attack could cause a considerable impact. Momentum is a botnet that was recently caught in the wild [54]. This botnet mainly targets vulnerable Linux devices that are susceptible to attacks. This botnet uses 36 DDoS attack vectors and Mirai, Bashlite, and Kaiten as backdoor variants for creating bots by exploiting vulnerable devices.

The 36 attack vectors that are used include the SYN flood, the DNS flood, and the UDP flood, and, as it is not so common for botnets, it also supports Memcached amplification. With so many devices connected to the net and with the amplification capabilities of Memcached-type services, this botnet could create many problems, even for well-resourced victims.

**Figure 6:**
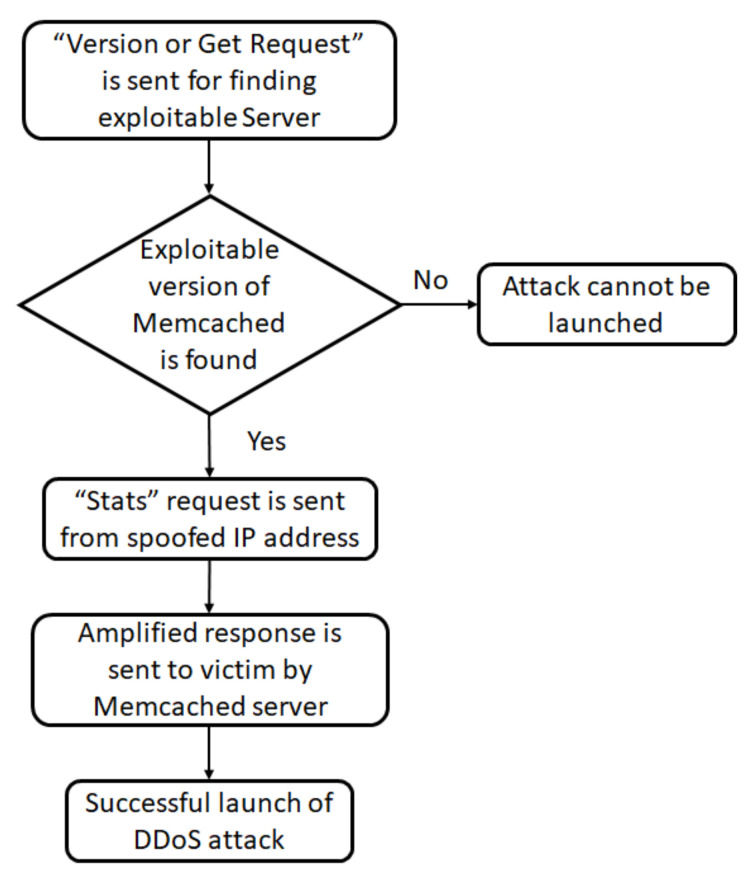
Process flow diagram of launching a DDoS attack using Memcached.

As shown in Figure 6, in a Memcached DDoS attack, the attacker sends a request to the Memcached server with a spoofed IP of the victim, so that the response is sent to the victim. Attackers deploy a two-step approach: the first step is finding exploitable Memcached servers, and the next step is to use them for an amplification attack.

## 5. Vulnerabilities of Memcached and Mitigation Techniques

To launch a Memcached DDoS attack, called a Memcrashed, attackers spoof the victim’s IP address to send queries to Memcached servers. This results in a significant data response from the Memcached server as it tries to help the victim. Nevertheless, in this process, all the victims’ resources get flooded by, typically, 50× responses. This server is designed to work with open connections, and it runs over the TCP/UDP port 11211. Memcached DDoS attacks can use open TCP and UDP ports at 11211, but the TCP port 11211 is not very vulnerable, as spoofing TCP queries is unreliable. Therefore, this attack usually exploits vulnerable Memcached servers with UDP enabled. It is considered by far the most elevated amplification attack.

Common Vulnerabilities and Exposures (CVE) is a system to provide a list of vulnerabilities and exposures in information security that are disclosed publicly. Some vulnerabilities that are related to Memcached servers, as published by CVE, are summarized in Table 2 [55].

The vulnerabilities exploited by attackers are as follows:

- UDP ports are enabled by default for Memcached versions up to 1.5.5, and the update or manual disabling of the port is required in these versions. Even after version 1.5.6, attackers can generate Memcached DDoS attacks, but the impact is reduced.
- Memcached architecture is such that the servers do not interact with each other; thus, if many requests are coming from the same source IP to all the servers, then no flags could be raised.
- In Memcached, there is no authentication of the client, as it only requires a key to function. This may cause trouble, as a simple key can lead to data stealing, and it also becomes easier to launch attacks.
- The Memcached server has a user-configurable limit for stored value; by default, this value is 1 MB. This value is user-configurable when under attack. It can be changed and exploited.
- Like UDP, unprotected DNS can also be used for amplification attacks, so vulnerabilities in regard to this should also be checked.
- A Memcached DDoS attack tool named Memcrashed is available online [56]; it is written in Python. These kinds of tools can create havoc, as even an inexperienced hacker can exploit vulnerabilities. It was seen in the past with Mirai that once the code was made public, many Mirai variants came into the public domain.

### Mitigation Techniques

Many cloud providers have laid down some best practices for users, which can curb Memcrashed to an extent. For example, the flush all/kill command was given as a solution, but this was widely rejected, as it contradicts the essence behind using Memcached servers.

- The most common and straightforward approach for this is blocking UDP/TCP port 11211 traffic.
- It has also been recommended not to use UDP frequently, and to keep it disabled by default.
- While using UDP, the response should be smaller than the request size; otherwise, there is always a chance of an amplification attack. UDP is a connectionless protocol and does not require authentication like the three-way handshake mechanism used by the TCP protocol for communication.
- The use of firewalls can always prevent DDoS attacks.
- Memcached is designed for private network use, so localhost binding with the help of a firewall can be of great help.

For this type of attack, it is necessary to understand that attackers are continuously looking for vulnerabilities, against which the system needs to respond quickly. The attack was first launched in 2018, and very soon, defenders found that several Memcached servers were exposed to public networks. Memcached was never supposed to be exposed to the public internet, but several Memcached servers were exposed to the net even after realizing this issue. To be precise, as revealed by Shodan, there are 77,561 exposed Memcached servers [57]. Not all of these may be vulnerable to DDoS attacks, but this is a considerable number. Given the capacity of an amplification factor as large as 51,200, as Cloudflare stated, even a small number of exploitable Memcached servers can cause a considerable impact. The proposed solution is applicable for service providers to ensure that such incidents of DDoS attacks do not take place in future. The solution will be useful for mitigating attacks regardless of compliance with best practices at the user end. With technological advancements, it becomes difficult for the user to be up-to-date. In this scenario, the proposed solution will be of great importance, as it puts the onus on the service provider.

## 6. Proposed Solution

Memcached attacks and their aftermath suggest that DDoS attacks using Memcached are the most critical unexplored cybersecurity dimension. As seen from previous works, there has been little to no research carried out on this dimension. This became the motivation behind the proposed method. Memcached is one of the most famous cache architectures because of its ease of use and flexibility. Some changes have been proposed to make Memcached architecture more secure. Memcached does not support encryption; thereby, it does not suffer from added overhead and time delays. This makes it a suitable choice for IoT nodes, which require a large amount of data to be handled alongside a higher data throughput. In [58], the authors proposed a new caching mechanism with encryption, and a 13% overhead was reported. This overhead cannot be accepted for latency-critical applications like healthcare and automated vehicles. 

As depicted in Table 3, several threshold-based mechanisms have been proposed by researchers in the past for detecting and mitigating DDoS attacks. It is evident from the literature that threshold mechanisms are efficient for the early detection of DDoS attacks [33,34,35,36]. The present work introduces context-aware computing to calculate the threshold and is applied to a volume-based DDoS attack. Consequently, to make Memcached secure, architectural change is proposed, so that the throughput is not compromised. Furthermore, the architectural change is designed with the provision that servers will not communicate if there is no suspicion of attack to ensure low latency while maintaining a near-comparable performance to Memcached.

### 6.1. Architectural Change in Memcached

Memcached attacks are some of the most severe volume-based DDoS attacks, and the solution for this is to divert the traffic [10]. In the undertaken study, this type of volume-based attack was detected by identifying a pattern using a threshold mechanism. Furthermore, in the conducted research, a change in the Memcached architecture is proposed, namely, a mechanism for communication between the servers in the case of suspicious activity. Usually, multiple requests are sent to many Memcached servers using the spoofed IP of the victim; so, duplicate requests arriving for a particular IP can be detected early on by communication between the different servers. Thus, the attack can be mitigated without a significant impact.

**Figure 7:**
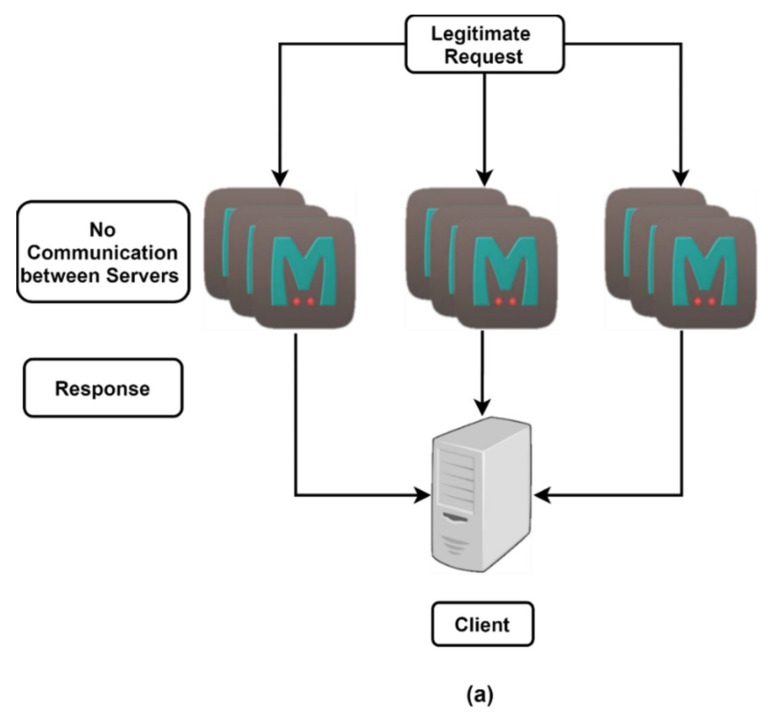

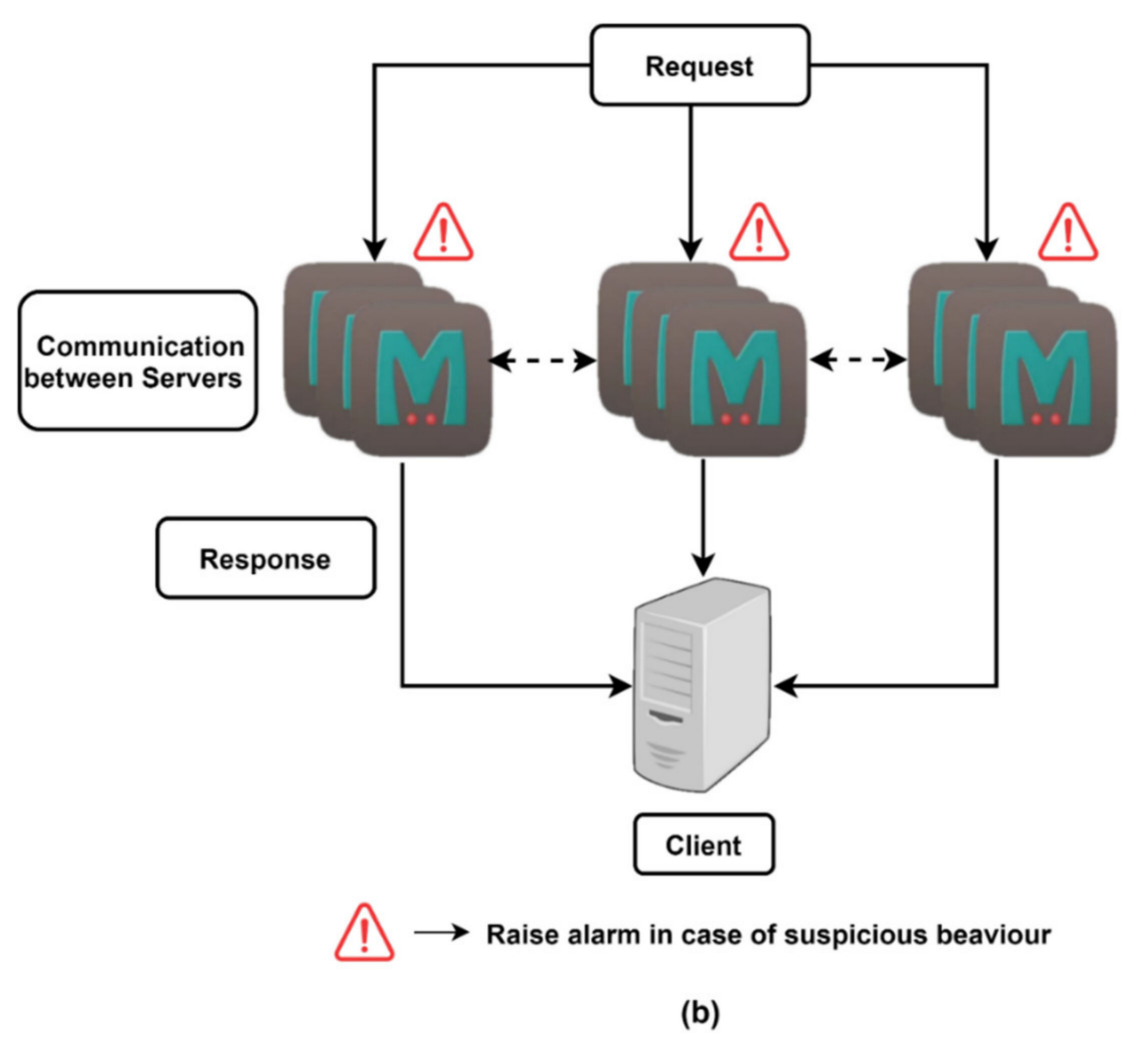
Architectural diagram for proposed setup, (**a**) before the threshold $n_{t}$, (**b**) after the threshold $n_{t}$.

Memcached was designed so that there would be no communication between the servers, to ensure low latency. Figure 7a represents a familiar scenario, in which there is no suspicious behavior. In these conditions, Memcached servers behave as originally designed and do not communicate with each other. The proposed architecture consists of a threshold for the number of requests beyond which the servers communicate, so that in typical scenarios, the working of Memcached remains as planned. The Memcached servers communicate, as depicted in Figure 7b, upon the suspicion of an attack. The suspected victim, i.e., the Memcached server for which the individual threshold value is crossed, sends SYN requests to the remaining Memcached servers to figure out whether or not an attack has occurred. The thresholds are synchronized between the servers, implying that $n_{t}$ is the same for all Memcached servers. Thus, the latency is only affected to a small extent in DDoS attack situations, where latency would be affected in any case. Alongside the updated Memcached server, context-aware computing is proposed to reduce the latency further. The optimal threshold value is to be decided by the service provider using context-aware computing, as it varies greatly depending on the service provider’s capabilities.

**Figure 8:**
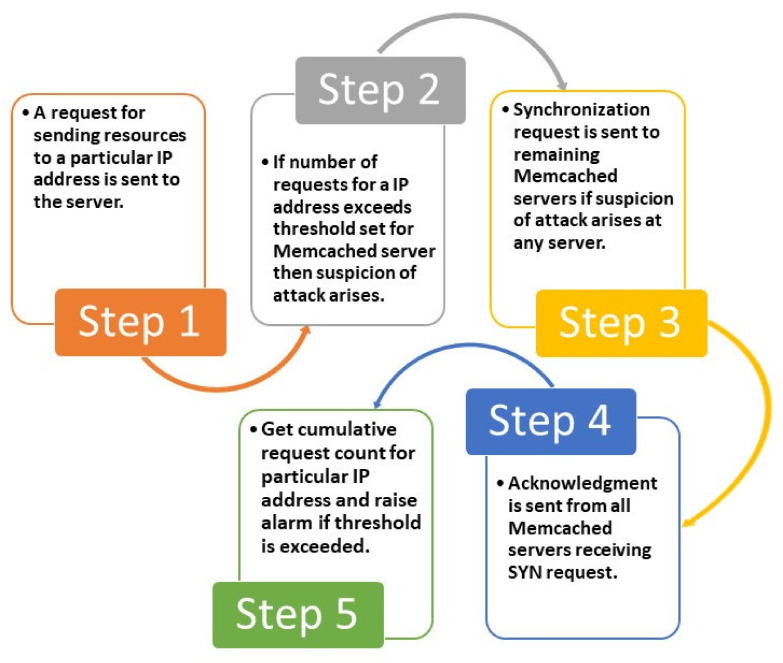
A detailed process flow of the proposed solution for DDoS attacks using Memcached servers.

In a DDoS attack, malicious actors send requests using IP spoofing to overwhelm the victim’s resources. The stepwise algorithmic representation is depicted in Figure 8. The proposed solution is an intrusion detection system (IDS) for raising the alarm at the time of intrusion. In an IDS, the focus remains on a higher recall rate, as detecting anomalous behavior is of priority in anomaly detection. The proposed system is able to raise the alarm for the service provider to take action. Human intervention is required for action; thus, the proposed solution does not slow down the system performance, in comparison to an intrusion prevention system (IPS). The solution is effective, as an alarm is raised even if one server is under attack, thus increasing the chances of mitigating an attack by early detection.

Step 1: A request for sending resources to a particular IP address is sent to the server, the record is kept as $n(IP_{i},\;\tau$), where, n is number of requests, $IP_{i}$ is IP address for the *i*th client, τ is time.

Step 2: For any Memcached server $\;m_{k}$, $k\;\in \;\left(1,\;N\right),\;$ where N is the total number of Memcached servers, suspicion of attack arises if for a particular IP address number of requests received at time τ is greater than the threshold value set for that Memcached server, i.e.,

(1) $$n(IP_{i},\;\tau )\;\geq \;n_{t}(IP_{i},\;\tau )\;$$

where, $n_{t}$—Threshold for the number of requests.

where $n_{t}$ is the threshold for the number of requests.

Step 3: If the conditions $n(IP_{i},\;\tau$) ≥ $n_{t}$($IP_{i},\;\tau$) holds good, then a Synchronization request (SYN) is sent to nearby/all Memcached servers,

(2) $$SYN\;\left(IP_{i}\right)$$

Step 4: All Memcached servers receiving SYN request then send the acknowledgement giving information about the number of requests for the suspected victim IP address for the said time duration, i.e.,

(3) $$ACK\;\left(n(IP_{i},\;\tau \right))$$

This scenario is depicted in Figure 7b, where communication between Memcached servers has been represented. All the variables, i.e., $n_{t},\;n_{at},\;\tau$ are arbitrary and can be updated according to requirements, thus enabling context-aware computing. 

Step 5: The cumulative request count is calculated as the sum of requests sent to the Memcached server $\;m_{k}$ for IP address of *i*th client and acknowledgement of requests received by other servers for the same client.

(4) $$\{\left(n(IP_{i},\tau \right){)}_{\;m_{k}}+\sum_{i}ACK\;\left(n(IP_{i},\tau \right))\}$$

The system is probable to be under attack if,

(5) $$\{\left(n(IP_{i},\tau \right){)}_{\;m_{k}}+\sum_{i}ACK\;\left(n(IP_{i},\tau \right))\}\geq n_{at}\left(IP_{i},\;\tau \right)$$

where $n_{at}\;$ is the cumulative threshold for the number of requests.

In this scenario, the alarm is raised for further investigation for the possibility of an attack. For any time duration τ, where ($\tau_{1}<\;\tau \;<{\;\tau }_{2}$), if any Memcached server sends a SYN request, no new SYN request from any other Memcached servers should be sent. The cumulative threshold is calculated for the whole duration ($\tau_{1}<\;\tau \;<{\;\tau }_{2}$); subsequently, any spike amounting to the threat coming in any Memcached server for the said duration will be automatically considered. SYN requests from Memcached servers can be sent for spikes after τ duration. The approach of sending one SYN request only for any time duration τ prevents performance degradation and maintains low latency and high throughput.

### 6.2. Case Study for Detecting DDoS Attack Using Memcached Servers

**Figure 9:**
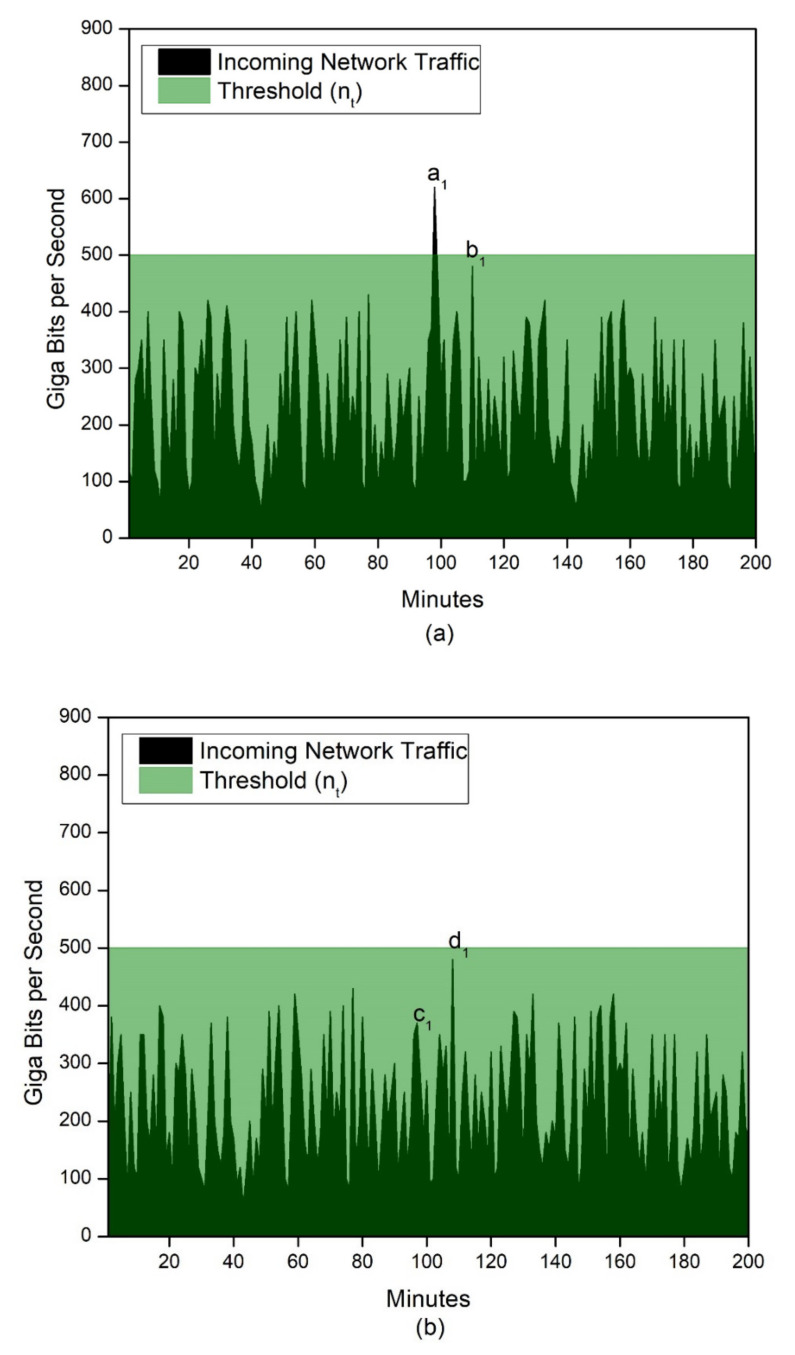

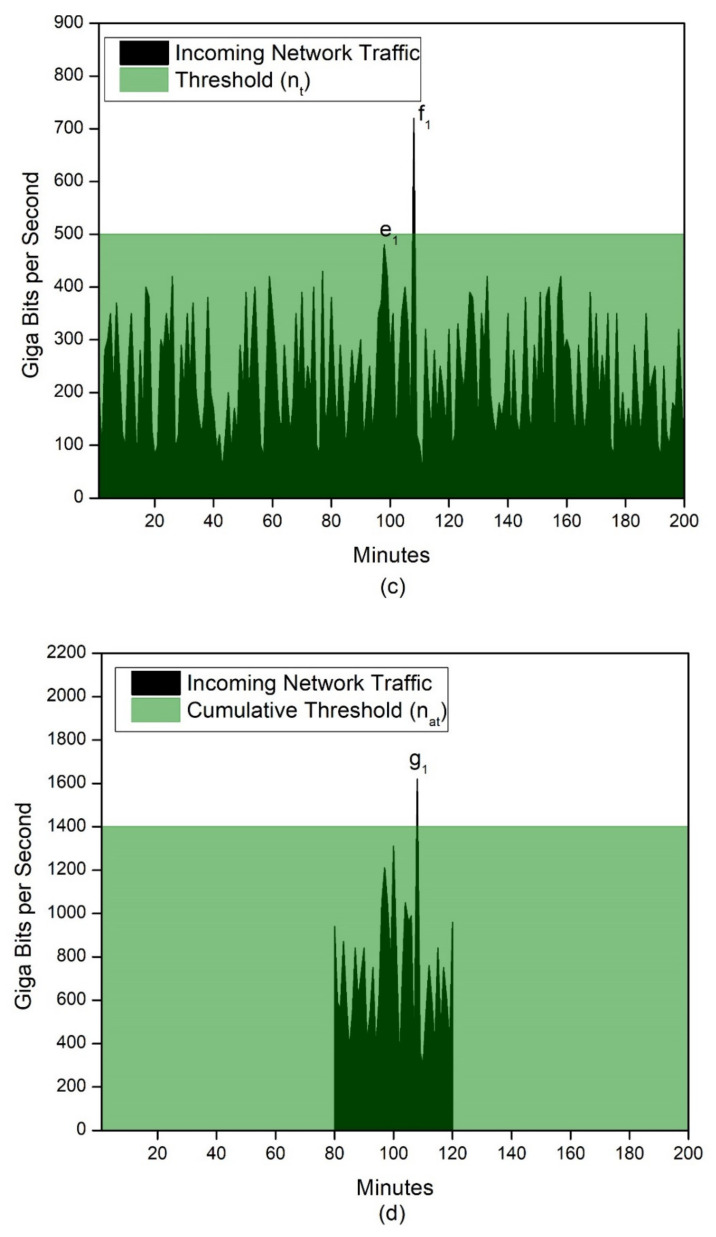
Case study of proposed solution for (**a**) Memcached server 1 with threshold value *n_t_*, (**b**) Memcached server 2 with threshold value *n_t_*, (**c**) Memcached server 3 with threshold value *n_t_*, and (**d**) cumulative result for raising the alarm with cumulative threshold value *n_at_*.

The experimental setup was created, as shown in Figure 7, on a Linux-based server on an Intel Core i7 dual-core processor, 16 GB RAM, 64-bit OS, using a virtual machine for capturing and storing the dataset. The proposed solution was analyzed using three Memcached servers with a realistic, representative dataset. As Memcached is intended for optimizing dynamic web applications, the threshold levels were defined using context-aware computing to provide optimum service without affecting the server performance. The effectiveness of the proposed solution is imperative, as it can be seen from the results that by proper selection of the threshold, pre-emptive action can be taken by raising the alarm in the case of a DDoS attack. For Memcached server 1, the threshold $n_{t}$ was exceeded at point a_1_ at the 100th minute, as shown in Figure 9a; at this stage, the SYN command was sent to all the Memcached servers for acknowledgement. As seen in Figure 9b, there was no spike in Memcached server 2. A spike at the 110th minute was seen for Memcached server 3, i.e., point f_1_ in Figure 9c, whereas the threshold was not exceeded at the 100th minute, when the SYN command was sent by Memcached server 1. This implied that the infiltration did not need to happen at the same time for all the servers. Figure 9d demonstrates the cumulative results obtained from all the Memcached servers for the time duration $\tau_{1}<\;\tau \;<{\;\tau }_{2}$. Since there was no SYN request beyond 80 <τ<120, no acknowledgement was sent, as is evident from Figure 9d, which shows nothing beyond this duration. The alarm was also raised at point g_1_ at the 110th minute, but not at the 100th minute, when the SYN request was raised initially. This implied that the proposed method avoided false positives and that the latency was not compromised, as the SYN and ACK requests were required only once for any time duration.

**Figure 10:**
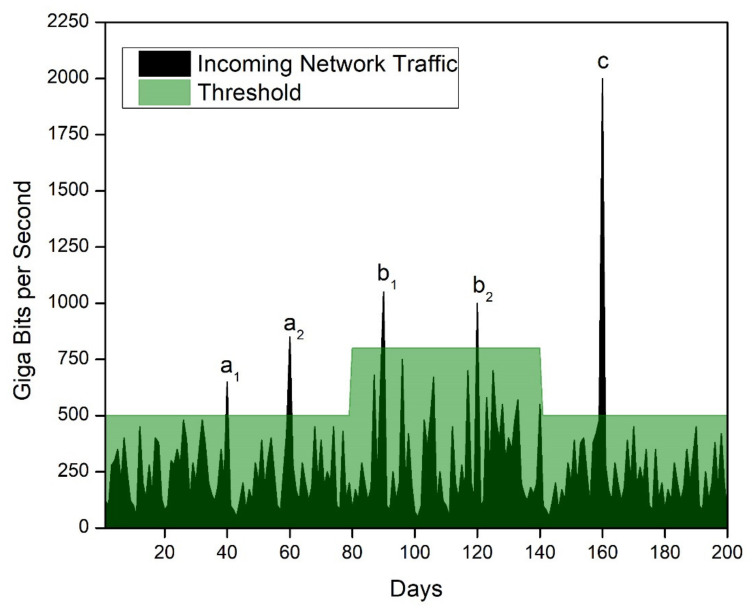
A graphical representation of proposed Memcached solution for multiple threshold levels, a_1_, a_2_, b_1_, b_2_, and c.

Before any attack, spurious activities are seen in the network traffic when the attacker tries to launch the attack. This is when an attack can be mitigated even before it happens, and before resources are lost. The proposed method can stop the attack before it can create trouble, if the variables are chosen carefully, as seen in Figure 10. In the figure, two threshold levels were chosen to indicate the differences between the threshold level choices. For example, if the threshold was selected to be at 500 Gbps, a_1_, a_2_, b_1_, b_2_, and c would be seen as suspicious activities, and an alarm could be raised for these values.

If 800 Gbps was selected as the threshold, a_2_, b_1_, b_2_, and c would be seen as suspicious activities, but an alarm would not be raised for a_1_. In this way, the threshold can be decided at the client level, and spurious activities are tracked. In the range of 80 to 140 days, several spikes could have been considered spurious for threshold value of 500 Gbps. Thus, using the proposed method for expected peak-traffic days, the client can decide to keep a higher threshold value, hence preventing a higher false-alarm rate. Figure 10 shows that c denoted an attack which could be stopped if an alarm were raised, using any threshold value. The proposed solution would not significantly impact the performance, since an SYN request would be sent to the other servers only once for any time duration if the threshold was crossed for multiple Memcached servers. This would be accomplished before an actual attack took place. The solution acts as a pre-emptive measure for detecting attacks; thus, it will enhance system performance at large.

## 7. Conclusions

Memcached is a useful model for managing large amounts of data, and it runs on several nodes with multiple cores. Its simplicity makes it a popular choice when working with IoT devices. Memcached does not support encryption; thereby, it does not suffer from added overhead and time delays. Memcached attacks and their aftermath suggest that DDoS attacks using Memcached are the most critical unexplored cybersecurity dimension. Devices using the IoT are also highly prone to DDoS attacks, due to the amplification capacity of Memcached. The kind of DDoS amplification attack (with the amplification factor being nearly 51,000) possible using Memcached servers makes them high-potential attack vectors, as already seen in the Momentum botnet attacks.

The possibility of a Memcached attack using botnets was surmised, and an approach was proposed for the early detection of such an attack. The presented solution considered architectural changes to the Memcached server to protect network service providers against DDoS amplification attacks. The methodology was designed so that, even if one Memcached server was under attack, it could be detected. Moreover, the performance of the servers would not be compromised by the proposed change. If multiple servers were suspected of being under attack, only one SYN request would have to be acknowledged by the other servers, and the attack could be identified. The use of context-aware computing for deciding the threshold increased the flexibility of the proposed solution.

As a future extension of this work, machine-learning techniques can decide which algorithm should be used to discard data in a particular application. Usually, LRU is used in Memcached, but dynamically deciding the data-discarding algorithms for each application can drastically improve the latency and throughput in diverse scenarios, and Memcached can work more effectively.

## Figures and Tables

**Table 1:** Analysis of architectural changes presented in the literature.

Reference	Year	Problem Statement	Architectural Change	Achievement
Lim et al. [14]	2013	Increased load on the network and database.	Authors introduced thin servers with smart pipes by coupling embedded low-power cores to the Memcached server, enabling GET requests to be processed in hardware.	Power–performance trade-off.
Lu et al. [15]	2014	Single-point failure mechanism of Memcached.	Authors proposed R-Memcached, where caches are replicated in the Memcached server.	Consistency among cache replicas.
Blott et al. [16]	2015	Limited value-store capacity in in-memory key-value stores such as Memcached.	A Hybrid of DRAM and serial-attached flash drive was proposed for increasing the value-store capacity.	High throughput and scalability.
Zaidenberg et al. [17]	2015	Data-discarding algorithm for Memcached.	In this work, five new algorithms were presented in place of the least-recently-used (LRU) algorithm for discarding data in Memcached.	Improved hit rate.
Singh et al. [20]	2018	Flaws in Memcached architecture and operations.	The authors identified flaws of Memcached architecture, and the prevention of DDoS attacks was also discussed.	Security steps for avoiding DDoS attacks.
Proposed work	2021	DDoS attack using Memcached.	Communication between Memcached servers is proposed in the undertaken study for detecting volume-based attacks.	High security from DDoS attacks while maintaining throughput latency.

**Table 2:** Detailed vulnerability description of Memcached.

Vulnerability Reference	Description
CVE-2020-10931	Insufficient authentication of user input is why this vulnerability exists in memcached.c when a binary protocol header is parsed in the try_read_command_binary() function. DoS attacks can be performed using this vulnerability.
CVE-2019-11596	“lru mode” and “lru temp_ttl” commands were found to be dereferencing the NULL pointer in Memcached versions before 1.5.14, making it prone to denial of service.
CVE-2019-15026	In Memcached version 1.5.16, while using UNIX sockets in memcached.c, a buffer over-read was found in conn_to_str, causing a denial of service.
CVE-2018-1000115	This is the vulnerability caused due to open UDP port at 11211. In UDP support up to Memcached version 1.5.5, network message volume could not be controlled sufficiently, making it vulnerable to denial-of-service attacks. An amplification factor of 50,000 could be achieved using this.

**Table 3:** Comparative analysis of proposed work with baseline techniques.

Author	Year	Applied Technique for Intrusion Detection in DDoS Attacks	IDS Applied for Detecting Attack Type	Remarks
Alamri et al. [33]	2020	Bandwidth control mechanism and XGBoost algorithm	DDoS attacks in Software-Defined Network	Trigger-based detection is applied using an adaptive-bandwidth-profile-based threshold where flawed flows are penalized for preventing bandwidth depletion.
Singh et al. [34]	2020	Threshold and entropy-based detection mechanism	Discriminating flash-crowd events from DDoS attacks	DDoS attacks on edge routers are detected using entropy and a threshold-based system.
Baskar et al. [35]	2021	Real-time traffic-monitoring algorithm using a multi-threshold system	Low-rate DDoS attacks	Low-rate DDoS attacks are detected using a multi-threshold traffic-analysis approach.
Jisa et al. [36]	2021	Threshold-based algorithm using network traffic parameter	Discriminating flash-crowd events from DDoS attacks	Dynamic threshold algorithm is introduced with less processing time for DDoS attack detection.
Proposed work	2021	Context-aware computing-based threshold mechanism	Memcached-based DDoS attacks	DDoS attacks using Memcached as an attack vector are mitigated efficiently by introducing architectural change in Memcached and using a context-aware threshold mechanism.

## Data Availability

Data will be made available on request.

## Abbreviations

**Terminology**	**Description**
IoT	Internet of Things
IIoT	Industrial Internet of Things
QoS	Quality of service
DDoS	Distributed denial of service
CPDoS	Cache-poisoned denial of service
IDS	Intrusion detection system
BAF	Bandwidth amplification factor
CoAP	Constrained Application Protocol
ARMS	Apple remote management services
TCP	Transmission Control Protocol
UDP	User Datagram Protocol
DNS	Domain name system
SNMP	Simple Network Management Protocol
WS-Discovery	Web Services Dynamic Discovery
SSDP	Simple Service Discovery Protocol
LDAP	Lightweight Directory Access Protocol
QOTD	Quote of the Day
NTP	Network Time Protocol
SOAP	Simple Object Access Protocol
RAM	Random-access memory
DRAM	Dynamic random-access memory
CV2X	Cellular vehicle-to-everything
CVE	Common Vulnerabilities and Exposures
LRU	Least recently used
FIFO	First in, first out
TLRU	Time-aware least recently used
ACK	Acknowledgement
SYN	Synchronize
Gbps	Gigabits per second
